# Polydatin inhibits cell proliferation, invasion and migration, and induces cell apoptosis in hepatocellular carcinoma

**DOI:** 10.1590/1414-431X20176867

**Published:** 2018-03-01

**Authors:** Yang Jiao, Yan Wu, Dong Du

**Affiliations:** 1 Department of Physical Examination, The Second Clinical College of Jinan University, Shenzhen People's Hospital, Shenzhen, China; 2 Department of Endocrinology, The Second Clinical College of Jinan University, Shenzhen People's Hospital, Shenzhen, China

**Keywords:** Polydatin, Hepatocellular carcinoma, Proliferation, Apoptosis, Wnt/beta-catenin

## Abstract

Polydatin, a small molecule from *Polygonum cuspidatum*, has many biological functions, particularly anti-cancer effects. However, the anti-cancer effects of polydatin in hepatocellular carcinoma (HCC) have not been examined yet. In the present study, MTT assay, BrdU assay, transwell invasion assay, and wound healing assay were performed to determine cell proliferation, invasion and migration. Flow cytometry and TUNEL assay were used to measure cell apoptosis. Quantitative real-time PCR and western blotting assays were used to determine mRNA and protein expression levels. Xenograft experiment was performed to determine the *in vivo* anti-tumor effect of polydatin. Immunostaining was performed to analyze the expression of caspase-3 and Ki-67. Our results showed that polydatin inhibited cell proliferation in a concentration-dependent and time-dependent manner in the HCC cell lines. Polydatin also induced cell apoptosis in a concentration-dependent manner possibly via increasing the caspase-3 activity, and up-regulating the protein expression of caspase-3, caspase-9, Bax, and down-regulating the protein expression of Bcl-2. In addition, polydatin treatment had an inhibitory effect on cell proliferation, invasion and migration in HCC cell lines. Polydatin treatment also suppressed the Wnt/beta-catenin signaling activities in HCC cells. Polydatin treatment significantly reduced tumor growth in nude mice inoculated with HepG2 cells, suppressed the expression of Ki-67, and increased caspase-3 expression and TUNEL activity. Our data indicated the important role of polydatin for the suppression of HCC progression.

## Introduction

Hepatocellular carcinoma (HCC) is a common primary malignancy of the liver and happens mainly in patients with chronic liver disease and cirrhosis. HCC is the third leading cause of cancer-related deaths worldwide (1). The standard treatment for HCC mainly involves liver transplantation, surgical resection and chemotherapy (2). Unfortunately, surgical resections are not suitable for HCC patients with advanced stage, especially with liver cancer metastasis (3). In addition, the current available chemotherapeutic drugs are not effective to treat advanced HCC (4). In this regard, it is necessary to find more effective compounds, which may provide novel therapy for HCC treatment, especially in the advanced stage.

With recent research in phytochemistry, the anti-cancer compounds from herbal plants are gaining interest, and studies have shown that about 50% of the small molecular anticancer drugs developed between 1950 to 2015 were from natural products or their derivatives (5,6). *Polygonum cuspidatum*, with its root and rhizome being commonly used, is a traditional Chinese herb, and has been listed in the pharmacopoeia for a long time (7). Studies have shown that one of the main compounds of *P. cuspidatum* is 3,4,5′-trihydroxystilbene-3-β-D-mono-D-glucoside (polydatin) (7). Previous studies have demonstrated a variety of biological functions of polydatin, including protecting against ischemia/reperfusion injury (8), congestive heart failure (9), endometriosis (10), and shock (11). Recently, the anti-cancer effects of polydatin have also been examined. For example, polydatin was found to induce apoptosis and inhibit growth of acute monocytic leukemia cells (12); polydatin also inhibited growth of lung cancer cells by inducing apoptosis and causing cell cycle arrest (13). In addition, polydatin promotes apoptosis through upregulating the ratio of Bax/Bcl-2 and inhibiting proliferation by attenuating the beta-catenin signaling in human osteosarcoma cells (14). Up to now, the role of polydatin in HCC has been not investigated.

For the first time in the present study, we examined the anti-cancer effects of polydatin in HCC cell lines (HepG2 and SMMC-7721) and in *in vivo* xenograft tumors, and also explored its potential underlying mechanism by using various molecular techniques.

## Material and Methods

### Cell lines and cell culture

Normal liver cell lines HL-7702, HCC, and liver cancer cell lines HepG2 and SMMC-7721 were purchased from the ATCC company (USA). All the cells were cultured in DMEM medium supplemented with 10% fetal bovine serum (FBS; Thermo Fisher Scientific, USA). The cells were kept in the humidified incubator at 37°C with 5% CO_2_. Polydatin was purchased from Sigma (USA).

### MTT assay for cell proliferation

HL-7702, HepG2, and SMMC-7721 cells were seeded on 96-well plates at a density of 10^4^ cells/well and cultured for 24 h. Then, the medium was replaced with DMEM or the same media containing different concentrations of polydatin (1, 3, 10, 30, and 100 µM). After further incubation for 24 or 48 h, MTT (Sigma) was added to each well of the 96-well plates, followed by a 4 h incubation. The medium was then discarded and 150 µL of DMSO was added into each well, and incubated for 20 min. The absorbance values at 490 nm were determined by a microplate reader (BioTek, USA).

### Flow cytometry for cell apoptosis analysis

For cell apoptosis analysis, Annexin V-FITC apoptosis detection kit (Abcam, UK) was used. Briefly, cells (HepG2 and SMMC-7721) were seeded at a density of 10^6^ cells/well in 12-well plates. After 48 h treatment with polydatin (1, 3, 10, 30, and 100 µM), the cells were washed with cold phosphate buffered saline, and then incubated with Annexin V-FITC/PI at room temperature for 5 min in the dark. The fluorescence of the cells was detected by flow cytometry by using a FITC signal detector and a PI signal detector (BD Biosciences, USA).

### Caspase-3 activity

The activity of caspase-3 was detected *in vitro* using a caspase-3 colorimetric assay kit (Abcam) according to the manufacturer's instructions. Briefly, cells (HepG2 and SMMC-7721) were seeded at a concentration of 10^6^ cells/well in 6-well plates, after 48 h treatment with polydatin (1, 3, 10, 30, and 100 µM), the cells were lysed and centrifuged at 12,000 *g* for 20 min at 4°C; resected tumor tissues were lysed and centrifuged at 12,000 *g* for 20 min at 4°C. The supernatant containing 50 µg of total protein were incubated with 5 µL caspase substrate in the 100 µL reaction buffer at 37°C for 1 h in the dark. The caspase-3 activity was determined by a microplate reader (BioTek) at 405 nm.

### Immunofluorescence of caspase-3

Cells (HepG2 and SMMC-7721) were treated with polydatin (30 µM) for 48 h and then fixed in 4% paraformaldehyde in FBS for 15 min. After fixation, the cells were permeabilized for 1 h in blocking buffer, and incubated with anti-caspase-3 antibody (1:250, Abcam) for 1 h at room temperature. Cells were then incubated with Alexa fluor-conjugated secondary antibody (1:500) for 1 h at room temperature. The nuclei were counter-stained with DAPI (Sigma).

### Quantitative real-time PCR (qRT-PCR) assay

Cells (HepG2 and SMMC-7721) treated with polydatin (30 µM) for 48 h were subjected to RNA extraction by using the Trizol reagent (Invitrogen, USA). Total RNA was reverse transcribed into cDNA by using the Reverse Transcription System Kit (Applied Biosystems, USA). QRT-PCR was performed with Applied Biosystems Prims 7500 Fast Sequence Detection System using SYBR Green master mix (Takara, China) according to the manufacturer's instructions. The relative mRNA expression levels of genes (DKK-1, beta-catenin, c-myc, cyclin D1, and survivin) were normalized to GAPDH, calculated by using the 2^-ΔΔCt^ method. All experiments were performed in triplicates.

### Western blotting assay

Cells (HepG2 and SMMC-7721) treated with polydatin (1, 3, 10, 30, and 100 µM) for 48 h were lysed for 30 min in cold lysis buffer. After centrifugation at 12,000 *g* for 5 min at 4°C, the supernatant was collected as the total cellular protein extracts. Protein samples were separated on the 10% SDS-PAGE, and then transferred onto a polyvinylidene difluoride membrane. Membranes were incubated with 5% skimmed milk in TBST at room temperature for 1 h. Then, the membranes were incubated with rabbit anit-caspase-3 (1:1500), rabbit anti-caspase-9 (1:1500), rabbit anti-Bax (1:2000), rabbit anti-Bcl-2 (1:1000), rabbit anti-DKK-1 (1:1500), rabbit anti-beta-catenin (1:2000), rabbit anti-c-myc (1:1000), rabbit anti-cyclin D1 (1:2500), rabbit anti-survivin (1:1000), and rabbit anti-GAPDH antibodies (1:3000) (all from Abcam) overnight at 4°C, and washed three times with TBST. Then, the membranes were further incubated with appropriate HRP-linked secondary antibodies. The bands of specific proteins were visualized by western blotting Luminal Reagent (Thermo Fisher Scientific) according to manufacturer instructions.

### BrdU assay

BrdU assay was used to determine cell proliferation of HCC cells (HepG2 and SMMC-7721) *in vitro.* The cells were seeded at a density of 10^4^ cells/well on 96-well plates. After treating with 30 µM polydatin or control medium for 48 h, cells were incubated with BrdU (20 µM) for 4 h. Cells were then permeabilized with 0.1% triton-100 in PBS and blocked with 3% FBS in PBS solution, and cellular DNA was denatured by DNaseI treatment. The incorporated BrdU was stained with Alexa Fluor¯488 anti-BrdU monoclonal antibody (BD Biosciences, USA). The nuclei were counter-stained with DAPI (Sigma).

### Transwell invasion assay

Transwell assay (Costar, USA) was used to determine cell invasion capacities of HCC cells (HepG2 and SMMC-7721) *in vitro*. The cells were seeded at a density of 10^6^ cells/well in 12-well plates. After treating with 30 µM polydatin or control medium for 48 h, HCC cells in 500 µL serum-free medium were seeded onto the upper chamber, coated with growth factor reduced Matrigel, and DMEM medium containing 10% FBS was added into the lower chamber as a chemoattractant. After further incubation, cells on the upper surface of the membrane were removed and the invading cells were fixed with 70% ethanol and stained with 0.5% crystal violet (Sigma). The number of invading cells were counted under a light microscope.

### Wound healing assay

HCC cells (HepG2 and SMMC-7721) were treated with 30 µM polydatin or control medium. After 48 h, cells were seeded in 6-well plates with 5×10^5^ cells/well and cultured till confluence. A wound was created by using a 100 µL pipette tip on the cell monolayer and images were taken at 0 h and 24 h to calculate the % of wound healing.

### Animals and *in vivo* tumor growth experiments

The male BALB/c nude mice were obtained from the Experimental Animal Central in Guangdong, kept in specific pathogen-free rooms, with free access to food and water. This study was carried out in strict accordance with the recommendations in the Guide for the Care and Use of Laboratory Animals of the National Institutes of Health. The protocol was approved by the Animal Ethics Committee of Shenzhen People's Hospital Protocol No. SZR2013J4). All efforts were made to minimize suffering.

Tumors were established by subcutaneous injection of 5×10^6^ HepG2 cells into the flanks of mice. Tumor volumes were estimated according to the formula: π/6 × a^2^ × b, where a is the short axis, and b the long axis of the tumor. When tumors reached 120 mm^3^ at about 2 weeks, the mice were randomly assigned into four groups with each group having 6 mice. The mice in the control group received a daily intraperitoneally (*ip*) injection of 100 µL of phosphate buffered saline, and the mice in other three groups received daily *ip* injections of 100 µL polydatin at doses of 25, 50, and 100 mg/kg. The tumor volume in the nude mice was measured every 4 days after the initial dose of polydatin. The mice were closely monitored and weighed. After 20 days of treatment, animals were euthanized and the tumors harvested for further analysis.

### TUNEL assay and Ki-67 immunostaining for tumor tissues

The resected tumor tissues were fixed in 4% paraformaldehyde for 24 h, embedded in paraffin, and sectioned in 5-μm thick sections for TUNEL assay and Ki-67 immunostaining. The terminal deoxynucleotidyl transferase-mediated deoxyuridine triphosphate nick-end labeling (TUNEL) assay was performed by using a commercially available kit (*In situ* Cell Death Detection kit, Roche, Switzerland). The TUNEL-positive cell nuclei were imaged at a magnification of 400×. For the immunostaining of Ki-67, the sections were subjected to antigen retrieval by boiling in 10 mM sodium citrate buffer for 15 min and blocked with goat serum at room temperature for 30 min. The sections were then incubated with anti-Ki-67 antibody (1:1000, Abcam) overnight at 4°C, and later incubated at room temperature with a biotinylated goat anti-rabbit secondary antibody working solution. After incubation with horseradish peroxidase-conjugated streptavidin, the sections were stained using 3, 3′-diaminobenzidine (Sigma) to reveal the antibody expression. The nucleus was stained with hematoxylin.

### Statistical analysis

Data were analyzed and plotted by using the Graphpad Version 6.0 (USA). All experiments were repeated at least three times. Data are reported as means±SE. Significant differences between groups were analyzed by the Student's *t*-test, one- or two-way ANOVA, as appropriate. P values less than 0.05 were considered to be statistically significant.

## Results

### Effects of polydatin on cell proliferation in normal liver cell lines and liver cancer cell lines

**Figure 1.**
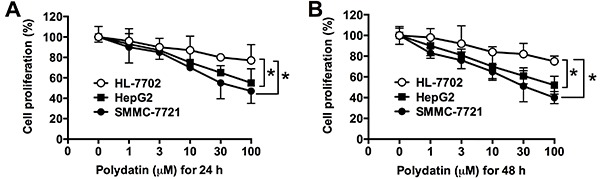
Effects of polydatin on cell proliferation in normal liver cell lines and liver cancer cell lines. *A*, Different concentrations of polydatin for 24 h, and *B*, for 48 h determined by MTT assay. Data are reported as means±SD. All experiments were repeated 3 times. *P<0.05 compared to control (HL-7702 group) (two-way ANOVA).

The MTT results showed that polydatin treatment with different concentrations (0, 1, 3, 10, 30, and 100 μM) for 24 and 48 h had no significant inhibitory effect on cell proliferation in HL-7702 cells. Furthermore, polydatin treatment in HepG2 cells and SMMC-7721 cells for 24 and 48 h significantly reduced the cell proliferation in a concentration-dependent manner. For HepG2 and SMMC-7721 cells, the inhibition on cell proliferation after 48 h treatment of polydatin was greater than 24 h treatment (Figure 1A and B).

### Effects of polydatin on cell apoptosis in liver cancer cell lines

**Figure 2.**
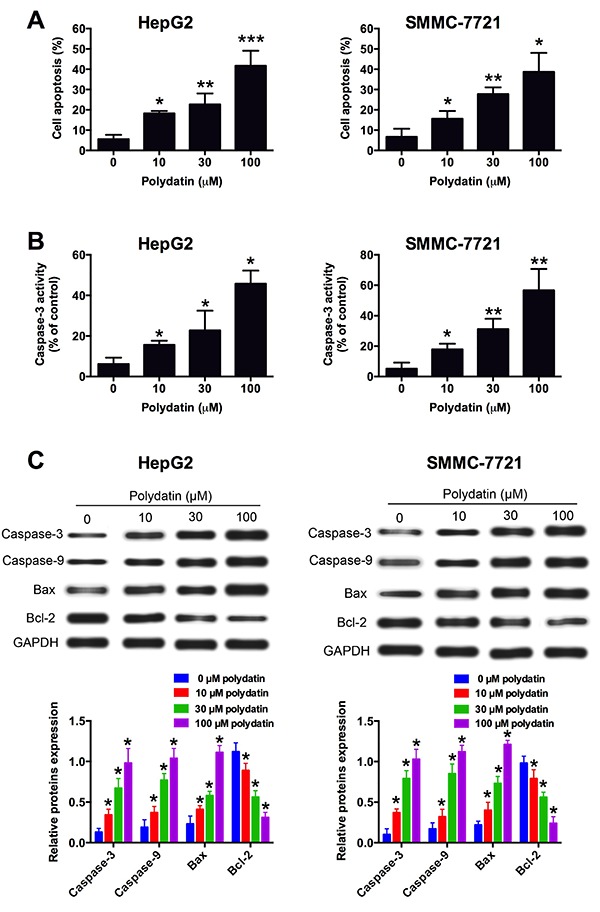
Effects of polydatin on cell apoptosis in liver cancer cell lines treated with different concentrations of polydatin for 48 h (*A*), determined by flow cytometry assay; *B*, effect of the treatment on caspase-3 activity measured by caspase-3 activity kit; *C*, protein expression levels of caspase-3, caspase-9, Bax, and Bcl-2 measured by western blotting assay. All experiments were repeated 3 times. Data are reported as means±SD. *P<0.05, **P<0.01, and ***P<0.001 compared to control group (one-way ANOVA).

The results showed that polydatin treatment for 48 h significantly increased the cell apoptotic rates in HepG2 and SMMC-7721 cells, and rates were associated with the increased concentrations of polydatin (0, 10, 30, and 100 μM; Figure 2A). To further confirm the mechanism of polydatin treatment on cell apoptosis, the caspase-3 activity was measured in HepG2 and SMMC-7721 cells after polydatin treatment. The results demonstrated that polydatin (0, 10, 30, and 100 μM) treatment for 48 h in HepG2 and SMMC-7721 increased the caspase-3 activity in a concentration-dependent manner (Figure 2B). The immunofluorescence results showed that polydatin (30 μM) treatment for 48 h significantly suppressed the expression of caspase-3 in HepG2 and SMMC-7721 cells (Supplementary Figure S1). In addition, the apoptosis signaling pathway analysis showed that polydatin treatment (0, 10, 30, and 100 μM) for 48 h in HepG2 and SMMC-7721 cells increased the protein expression of caspase-3, caspsase-9, and Bax, and suppressed the protein expression of Bcl-2 (Figure 2C).

### Effects of polydatin on cell proliferation, invasion and migration in liver cancer cell lines

**Figure 3.**
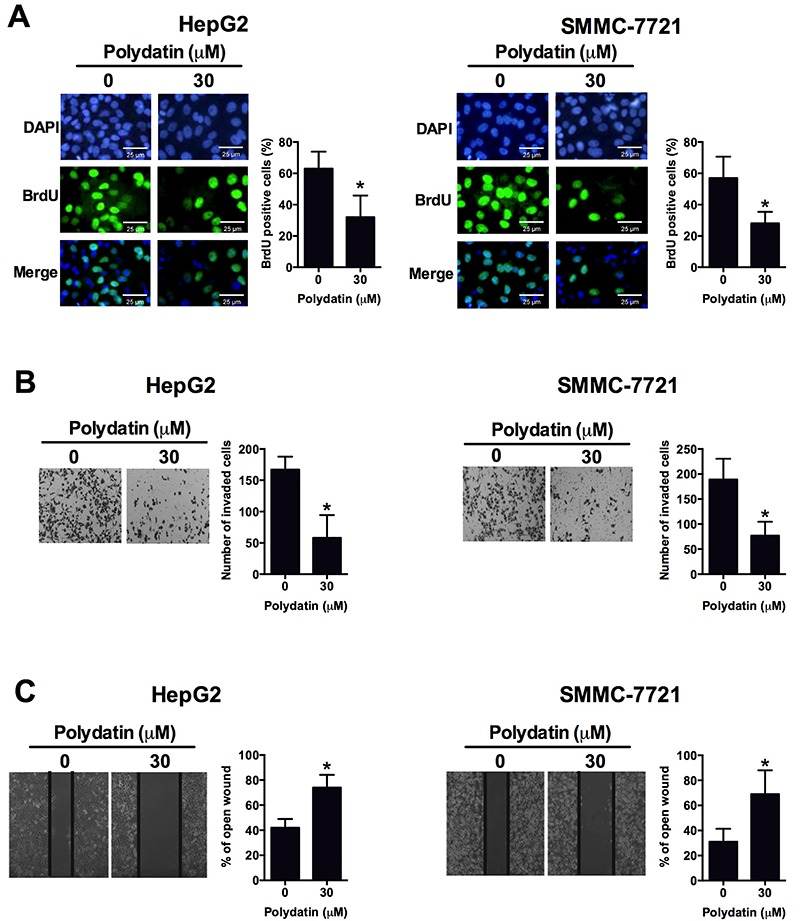
Effects of polydatin-treated liver cancer cell lines HepG2 and SMMC-7721 on *A*, cell proliferation determined by BrdU assay; *B*, cell invasion determined by transwell cell invasion assay; *C*, cell migration determined by wound healing assay. Data are reported as means±SD. All experiments were repeated 3 times. *P<0.05 compared to control group (Student's *t*-test).

Polydatin (30 μM) treatment for 48 h significantly reduced the percentage of BrdU positive cells when compared to control (Figure 3A). Polydatin (30 μM) treatment for 48 h also significantly reduced the invaded cell number as determined by the transwell cell invasion assay in HepG2 cells and SMMC-7721 cells when compared to control (Figure 3B). Furthermore, the same treatment delayed the wound healing rate in HepG2 cells and SMMC-7721 cells compared to the control group (Figure 3C).

### Effects of polydatin on the Wnt/beta-catenin signaling activity

**Figure 4.**
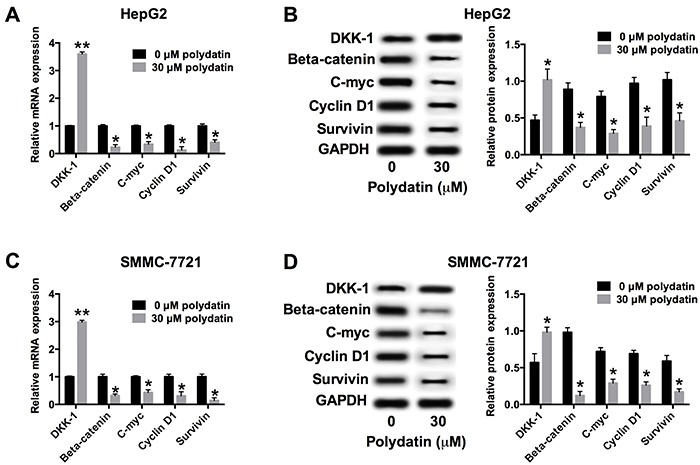
Effects of polydatin on Wnt/beta-catenin signaling activity in liver cancer cell lines. *A*, mRNA expression levels and *B*, protein levels of DKK-1, beta-catenin, c-myc, cyclin D1, and survivin in HepG2 cells determined by qRT-PCR and western blot assay, respectively; *C*, mRNA expression levels and *D*, protein levels of DKK-1, beta-catenin, c-myc, cyclin D1, and survivin in SMMC-7721 cells. Data are reported as means±SD. All experiments were repeated 3 times. *P<0.05, **P<0.01 compared to control group (Student's *t*-test).

Polydatin (30 μM) treatment for 48 h significantly suppressed the mRNA and protein expression of beta-catenin, c-myc, cyclin D1, and survivin, and increased the mRNA and protein of DKK-1 in HepG2 cells (Figure 4A and B). Similarly, the same treatment also had similar effects on the Wnt/beta-catenin signaling in SMMC-7721 cells (Figure 4C and D).

### Effects of polydatin on *in vivo* tumor growth

**Figure 5.**
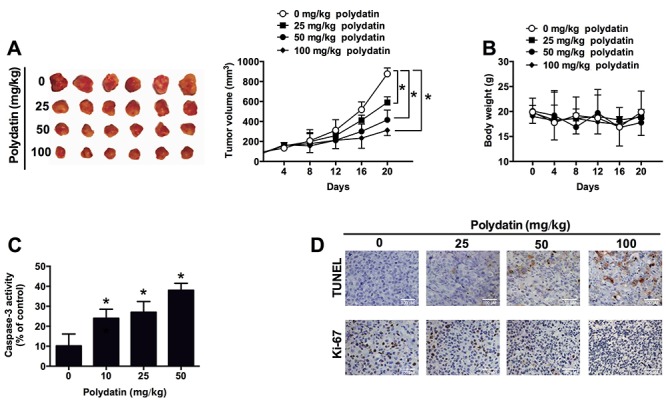
Effects of polydatin on tumor growth in the nude mice inoculated with HepG2 cell lines. *A*, tumor volume; *B*, body weight; *C*, caspase-3 activity from the resected tumor tissues; *D*, cell apoptosis and cell proliferation detected by TUNEL assay and Ki-67 immunostaining in the resected tumor tissues. All experiments were repeated 6 times. Data are reported as means±SD. *P<0.05 compared to control group (two-way ANOVA for tumor volume, one-way ANOVA for caspase-3 activity).

The effect of polydatin on *in vivo* tumor growth was examined in the nude mice inoculated with HepG2 cells. Polydatin treatment (25, 50, and 100 mg/kg) significantly reduced the tumor growth in the nude mice compared to the control group in a dose-dependent manner (Figure 5A). The treatment of polydatin had no effect on the body weight (Figure 5B). In addition, we also found that polydatin treatment significantly increased the caspase-3 activity in the resected tumor tissues compared to the control group, and the effect was also dose-dependent (Figure 5C). Furthermore, the results of the TUNEL assay and Ki-67 immunostaining for assessment of cell apoptosis and cell proliferation showed that polydatin treatment dose-dependently increased the TUNEL activity, and suppressed the expression of Ki-67 in tumor tissues (Figure 5D).

## Discussion

Polydatin is a glycoside of resveratrol, and the glycoside group is bonded in the C-3 position by substituting a hydroxyl group, which results in changes in its biological properties (15). Studies have shown that polydatin is more efficiently absorbed and more resistant to enzymatic oxidation than resveratrol (16). Based on previous studies, the anti-tumor effect of polydatin has been shown in various types of cancer cell lines. Polydatin can inhibit cell proliferation via attenuating the β-catenin signaling and promotes cell apoptosis via up-regulation the ratio of Bax/Bcl-2 in osteosarcoma (14). Polydatin also induces apoptosis and inhibits growth of acute monocytic leukemia cells (12). Polydatin was found to exhibit anti-growth activity against 3D cell aggregates of the SKOV-3 and OVCAR-8 ovarian cancer cell lines (17). In addition, polydatin also inhibits cell proliferation by inducing apoptosis and cell cycle arrest in lung cancer cell lines and colorectal cancer cell lines (13,18). Our data showed that polydatin was effective in inhibiting cell proliferation in a concentration-dependent manner. Taken together, these results may suggest the anti-tumor role of polydatin in HCC.

Apoptosis, also known as programmed cell death, is morphologically characterized by cell shrinkage, membrane remodeling, cell blebbing, chromatin condensation, and DNA fragmentation with apoptotic bodies (19). The induction of apoptosis is suggested to be a good strategy in cancer treatment (20). Importantly, polydatin effectively induced cell apoptosis in various cancer cell lines including osteosarcoma cells, acute monocytic leukemia cells, lung cancer cells and colorectal cancer cells (12–14,18). In the present study, the data consistently showed that polydatin induced cell apoptosis in a concentration-dependent manner. Further mechanistic findings showed that polydatin increased caspase-3 activity and also increased the protein expression levels of caspase-3, caspase-9, and Bax, and decreased the protein expression levels of Bcl-2. These results suggested that polydatin promoted apoptosis in HCC cells, which may contribute to inhibitory effects of polydatin on cell proliferation.

Cancer cell invasion and migration is believed to largely contribute the cancer metastasis (21), and our results showed that polydatin also inhibited HCC cell invasion and migration measured by transwell invasion assay and wound healing assay, respectively. However, the mechanisms underlying polydatin inhibiting HCC invasion and migration were not examined. Aberrant activation of Wnt/beta-catenin signaling has been shown to be associated with pathogenesis of HCC (4,22,23). In the present study, we found that polydatin treatment significantly suppressed the activity of Wnt/beta-catenin signaling in HCC cell lines, which was consistent with a previous study showing that polydatin attenuated the Wnt/beta-catenin signaling in human osteosarcoma cells (14). For the *in vivo* studies, we demonstrated the anti-tumor growth effect of polydatin in the nude mice inoculated with HepG2 cells. The anti-tumor growth effect might be through the promotion of apoptosis and suppression of proliferation, as polydatin treatment increased the caspase-3 activity and TUNEL activity, and decreased the expression of Ki-67 in the resected tumor tissues. Up to now, the *in vivo* anti-tumor effect of polydatin was largely unknown. As polydatin is a glycoside of resveratrol, the *in vivo* anti-tumor effect of polydatin might be similar to resveratrol. Indeed, the treatment of resveratrol in animal models has been shown to have a protective effect against tumor growth via different mechanisms (24). Therefore, it is necessary to investigate the possible mechanisms for the *in vivo* anti-tumor effect of polydatin.

In summary, the present study demonstrated that polydatin inhibited cell proliferation, invasion, migration, and induced cell apoptosis in HCC cells; it also exhibited *in vivo* anti-tumor activity. Our data suggest an important role of polydatin in suppressing HCC progression.

## Supplementary material

Click here to view [pdf].
